# ‘Single-Seed-SpeedBulks:’ a protocol that combines ‘speed breeding’ with a cost-efficient modified single-seed descent method for rapid-generation-advancement in oat (*Avena sativa* L.)

**DOI:** 10.1186/s13007-023-01067-1

**Published:** 2023-08-27

**Authors:** Milcah Kigoni, Megan Choi, Juan David Arbelaez

**Affiliations:** Department of Crop Sciences, University of Illinois, Turner Hall AW-101, 1102 S. Goodwin Ave., 046, Urbana- Champaign, Urbana, IL 61801 USA

**Keywords:** Oat, *Avena sativa*, Speed breeding, Single-seed descent, Plant breeding, Rapid-generation-advancement, Small grains

## Abstract

**Background:**

In plant breeding, one of the most cost-effective and efficient ways to increase genetic gain is to reduce the breeding cycle time. In general, modern breeding methods for self-pollinated crops should strive to develop fixed lines at the lowest possible cost and in the minimum possible amount of time. Previous studies on spring oat (*Avena sativa* L.) showed that combining high plant density with limited soil fertility and moisture levels in a growth media like sand effectively decreases the time and cost of generating fixed single-seed descent lines. More recently, ‘speed breeding,’ or the exposure to prolonged photoperiod regimes of 22 h, has been shown to decrease flowering time in oat significantly. The goal of this study was to combine ‘speed breeding’ with high-density planting in a limited soil fertility media to reduce further the costs and time required to develop oat single-seed-descent lines.

**Results:**

We grew oat plants at low density in potting-mix (control), high density in potting-mix (HD-soil), and high density in sand (HD-sand) under 16 and 22 h of day length. We observed that oat plants grown in HD-sand and exposed to 22 h day length reduced their flowering time by around 20 and 5 days on average compared to those grown in control conditions at 16 and 22 h, respectively. We also observed that 85% of plants grown at high density in sand produced a single seed when grown in bulk conditions. In contrast, only 40% of plants grown at high density in potting-mix produced a single seed.

**Conclusions:**

Our novel protocol showed that oat plants grown in high-density bulks, using sand media and 22-hour day length, reduced their flowering time by 20 days compared to control conditions and produced plants with single seeds, following closely single-seed descent assumptions while significantly reducing labor costs and greenhouse space. This methodology can be deployed in oat breeding programs to help them accelerate their rate of genetic grain for multiple traits.

**Supplementary Information:**

The online version contains supplementary material available at 10.1186/s13007-023-01067-1.

## Introduction

Innovations in plant breeding methods and strategies have significantly shaped our current global food production [1]. However, climate change, rapid demographic growth, reductions in arable land, and localized malnutrition are intrinsic challenges that have accelerated the demand for better-adapted, high-yielding, and more nutritious crop varieties [2–4]. To address these issues, plant breeding programs must integrate novel strategies that include new technologies, tools, and methods to fast-track genetic gain and identify superior lines for variety release.

Oat (*Avena sativa* L.) is a self-pollinated cereal crop grown for grain consumption, animal feed, forage, and as a cover crop across temperate regions worldwide [5–7]. Improving the production of multi-purpose crops, such as oat, is a practical approach to addressing our current demands for nutritious food and improving the sustainability of agricultural systems. Conventionally, to complete one breeding cycle in oat, an average of six years is required. A typical oat breeding cycle involves four stages: crossing, generation of lines, phenotypic evaluation, and finally, selection of lines for crossing [8]. The time required to generate lines can be reduced using off-season rapid-generation-advancement techniques [8, 9]. In oat, the time required to go from seed to seed during the inbreeding process determines the duration of the line generation stage. Methods that enhance rapid and more efficient growing protocols at the line generation stage can significantly accelerate variety development and increase the rate of genetic gain [10].

Breeding methods such as single-seed descent (SSD) [11] can be carried out using rapid-generation-advancement techniques to speed up the generation of nearly homozygous lines. In SSD, one seed from each segregating plant within a family is harvested and advanced. This process is repeated through multiple generations to achieve homozygous breeding lines [11, 12]. Single-seed descent is a practical and straightforward approach that enables the genetic variation spectrum within families to be represented and evaluated in homozygous plants [13]. Single-seed-descent also minimizes natural selection and sample biases [14–16]. Traditional SSD methods grow individual plants separately, and one seed per plant is harvested to avoid population unbalances in the next generation. Growing multiple individual plants in single pots from each family can require extensive indoor spaces, and individual harvesting seeds from each family requires more labor when compared with family mass or bulk protocols. In bulk protocols, segregating populations or families are grown in one or a few pots, harvested, and threshed together. Bulk methods simplify logistics and save resources. Unfortunately, when families are advanced in bulk, segregating genotypes may not be equally represented in each generation. Some individuals produce more seeds than others, ultimately reducing genetic variation among the final set of lines developed. In oat, the family bulk method has been modified to drastically increase the number of plants producing single seeds, following SSD assumptions while saving resources and labor time. Grafius [17] illustrated that growing oat plants in near-starvation conditions cause plants to produce few seeds. Subsequently, Cisar et al. [18] developed a modified SSD (mSSD) protocol, showing that growing plants at high-density seeding rates in a limited nutrient and water media, like sand, produced, on average, 84% of plants to be single-seeded. With this protocol, a family of 100 breeding lines can be grown in a single 15 cm pot and harvested in bulk instead of individually planting and harvesting 100 small pots. The mSSD reduced space and labor requirements while producing similar results to SSD.

Single-seed descent protocols have surged in popularity across many crops due to the ability to rapidly advance lines for multiple generations per year under controlled environmental conditions through ‘speed breeding’ [9, 17, 18]. Speed breeding uses prolonged photoperiods to accelerate the developmental rate of plants [18]. Extending the photoperiod to 22 h a day is one of the main techniques used to accelerate growth in speed breeding protocols [9, 18]. Experiments that have evaluated speed breeding protocols in oats have shown that flowering time can be reduced by 11 days when plants are grown under 22 h of continuous light compared to 16 h of day length [19].

While significant advances have been made toward implementing speed breeding in oat, no studies to our knowledge have reported the effects of combining speed breeding with Cisar’s mSSD protocol to reduce generation intervals while minimizing labor and space requirements [11]. This study aims to optimize a protocol that combines the benefits of speed breeding and bulk growing conditions while achieving results similar to SSD. To do this, we developed a system for rapidly accelerating the generation turnover rate while maximizing the number of plants that produce a single seed. In developing this system, we *i*) identified the combination of high seeding density, growth media, and photoperiod regimes that produce the highest percentage of plants with single seeds in the shortest time used in this study and *ii*) developed a protocol for generating new oat lines in 1-year using the University of Illinois Spring Oat breeding program as a case example. These findings will allow temperate Spring small grain breeding programs to take advantage of speed breeding while saving time and resources using bulk growing methodologies.

## Materials and methods

### Plant material

For the experiments in this study, we used the spring oat cultivars Ogle, Spurs, Buckskin, Saber, Reins, Deon, Excel, and Colt. These eight varieties were widely evaluated in the Illinois Oat Variety Testing trial and showed a wide range of phenotypic variance in agronomical and phenological traits (Supplemental Table 1). Data collected from 2019 to 2021 at the University of Illinois Research and Educational Center in Urbana and the Northwestern Illinois Agricultural Research and Demonstration Center in Monmouth, Illinois, showed that the yield of these cultivars ranged from 3,514 to 4,052 kg/ha, flowering date expressed in Julian days ranged from 160 to 167. Plant height ranged from 86 to 100 cm (Supplemental Table 1). These lines represent 34 years of breeding and released cultivars in the Midwest U.S.A.

### Growth conditions

We tested the effects of two lighting treatments and three soil-media/seeding-density treatments and their interactions on the length of the growth cycle and the proportion of plants that produce a single seed [11, 12]. The two lighting treatments were 16 and 22 h of light. At the University of Illinois, we have used 16 h (16 h.) of supplemental lighting to grow oat plants optimally in greenhouses. The 22 h of light treatment (22 h.) is the standard protocol for speed breeding for small grains [9, 20]. From now on, we will define the 22-hour light treatment as speed breeding. The soil-media/seeding-density treatments were low-density in a potting-mix media (Control), high-density/potting-mix-media (HD-soil), and high-density/sand-media (HD-sand). The Control method is considered optimal for plant health and vigor. The HD-sand treatment is used in the modified single-seed-descent (mSSD) protocol reported by Cisar et al. [18] and implemented at the University of Illinois at Urbana-Champaign (UIUC) for generation advancement.

#### Factor 1 Supplemental lighting: with and without speed breeding

**Fig. 1:**
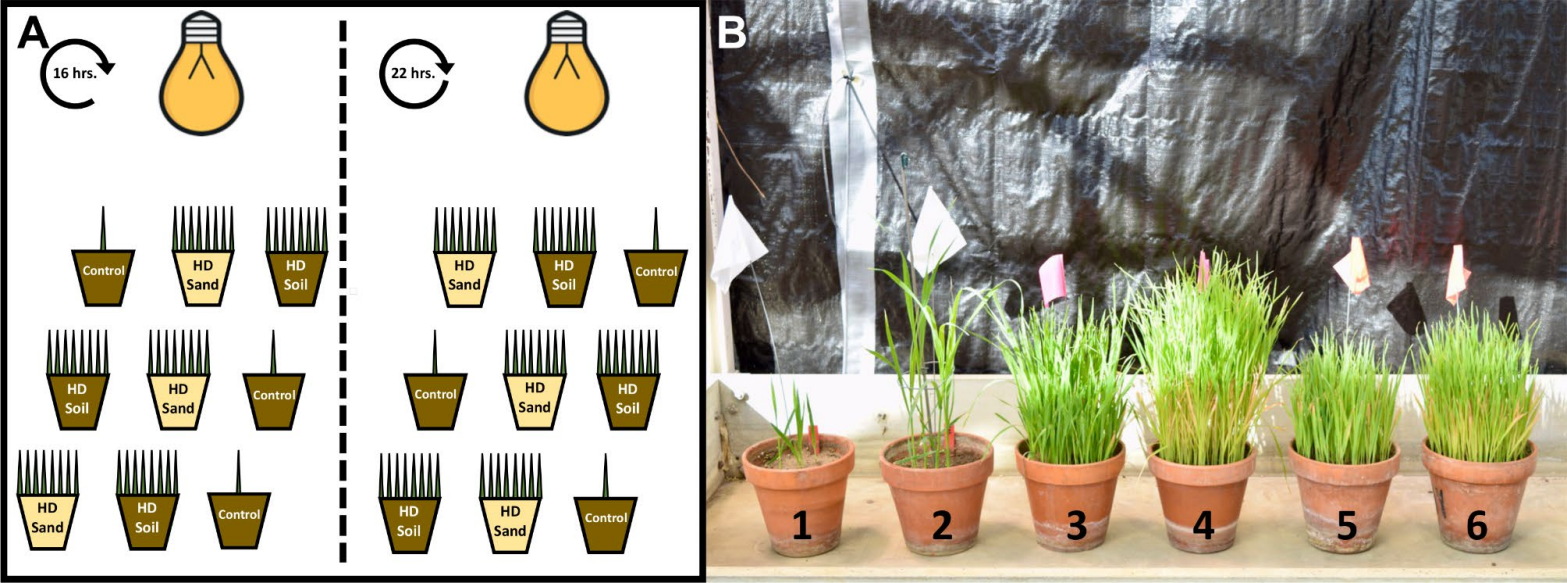
Growth conditions and experimental design. **A)** Representation of the experimental design showing the two-way factorial design with the two factors considered were: two lighting treatments (16- and 22-hrs. of supplemental lighting), and three soil media/seeding-density treatments (Control, HD-soil, and HD-sand). ^a^ Oat plants grown at 16 (1, 3, and 5) and 22-hrs. (2, 4, and 6) of supplemental lighting in different soil/media, including Control (1 and 2), HD-soil (3 and 4), and HD-sand (5 and 6)

We conducted the experiment in this study at the Plant Care Facility (PCF) greenhouses at UIUC between February 12 and April 30, 2021. We used two greenhouse rooms alongside each other, where we maintained the temperature conditions at 23 ºC during the day and 21 ºC at night. In one room, we supplemented light for 16 h (16 h.) or without speed breeding, and in the other room, we kept lighting for 22 h (22 h.) or with speed breeding (Fig. 1). Lighting was provided by using standard metal halide lamps. Light intensity at bench level in both rooms was approximated to 900 µE^− 2^s^− 1^.

#### Factor 2 growth media and planting density: modified SSD method

To generate three soil-media/seeding-density treatments for this study, we used two growth media types and two seeding densities that we combined to create three different treatments. The grow media we used were washed torpedo sand and a potting-mix. The potting-mix medium is more available and affordable than sand. It comprises one part peat, one part soil, and one part torpedo sand. The first seeding density we used was optimal or low seeding density, where we planted five seeds in six-inches-clay-pots filled with approximately 12 cm of media. The second seeding density we used was a high-seeding density treatment where we planted 150 seeds in six-inches-clay-pots filled with about 12 cm of media. The three final growing media/seeding-density treatments we used were low seeding density in potting-mix (Control), high seeding density in potting-mix (HD-soil), and high seeding density in the sand (HD-sand) (Fig. 1). The latter is the modified single-seed descent (mSSD) method implemented by Cisar et al. [18]. Fertilization was done only in Control pots by adding one teaspoon of Scotts Miracle-Gro Company’s Osmocote Smart-Release Plant Food, flower & vegetable (polymer-coated: Ammonium Nitrate, Ammonium Phosphate, Calcium Phosphate, and Potassium Sulfate all coated to give 12% slow-release Nitrogen (N), 12% Available Phosphate (P_2_O_5_) and 12% Soluble Potash (K_2_O)). Plants were watered daily, and three applications of safer soap were used to control aphid infestations.

### Experimental design, data collection, and analysis

We grew the eight oat varieties in a randomized, replicated two-way factorial design with three replications. The two factors considered were: *i*) two lighting treatments (16- and 22 h. of supplemental lighting), and *ii*) three soil media/seeding-density treatments (Control, HD-soil, and HD-sand) (Fig. 1). We planted a total of 144 experimental units or pots in this study.

We collected phenotypic data on flowering time, plant height, and the number of seeds per panicle using the Fieldbook mobile application [21]. Flowering time (FLW) was defined as the number of days from planting to 50% full panicle emergence. Plant height (PH) was measured at maturity as the distance from the soil surface to the panicle tip in centimeters. The number of seeds per panicle was measured in the high-density seeded treatments by taking 30 plants randomly in each pot and counting the number of seeds. Phenotypic data is available in Supplemental Table 1.

To analyze the effects of each factor on FLW and PH, we used the following linear model:

$$\begin{gathered}{y_{ijkl}} = \mu + {S_i} + {P_j} + {G_l} + {B_{k\left( i \right)}} + S{P_{ij}} + S{G_{il}} + P{G_{jl}} \hfill \\\,\,\,\,\,\,\,\,\,\,\,\,\,\,\,\,\,\,\,\, + SP{G_{ijl}} + {e_{ijkl}} \hfill \\ \end{gathered}$$,

where ${y}_{ijkl}$ is the response variable (FLW, or PH) in the *i*-th light treatment, *j*-th media/seeding-density, *l*-th genotype, and *k*-th block; $\mu$ is the overall mean, ${S}_{i}$ is the main effect of the *i*-th light treatment (i.e., 16- or 22-hrs.), ${P}_{j}$is the main effect of the *j*-th media/seeding-density (i.e., Control, HD-soil, or HD-sand), ${G}_{l}$ is the main effect of the *l*-th genotype (oat cultivar), ${B}_{k\left(i\right)}$ is the main effect of the *k*-th block nested in the *i*-th light treatment, ${SP}_{ij}$ is the interaction effect between the *i*-th light treatment and the *j*-th media/seeding-density, ${SG}_{il}$ is the interaction effect between the *i*-th light treatment and the *l*-th genotype, ${PG}_{jl}$, is the interaction effect between the *j*-th media/seeding-density and the *l*-th genotype, ${SPG}_{ijl}$ is the interaction effect between the *i*-th light treatment, *j*-th media/seeding-density, and the *l*-th genotype, and ${e}_{ijkl}$ is the experimental error at the experimental unit. Here e_ijkl_~N(0,𝜎). For the number of seeds per plant, we randomly harvested 30 plants per pot and counted the number of seeds in each plant. We used the number of plants with a single seed as the response variable in a linear model with the same parameters described in this section. We estimated multiple comparisons using Tukey’s HSD (honestly significant difference) method [22]. For each trait, we estimated broad-sense heritability of genotypes means, *H*^*2*^, using the formula of [23] as follows: ${H}^{2}={\sigma }_{G}^{2}/({\sigma }_{g}^{2}+\left(\frac{{\sigma }_{GE}^{2}}{E}\right)+(\frac{{\sigma }_{e}^{2}}{Er}\left)\right)$. With ${\sigma }_{g}^{2},{\sigma }_{GE}^{2}$ and ${\sigma }_{e}^{2}$, the genotype, genotype by the environment, and error variance components, respectively, with *E* and *r* the number environment and replicates. We used the statistical software R [24] and the packages lme4 [25] and agricolae [26] to analyze the data generated in this study.

## Results

### The effect of lighting and media/seeding-density in flowering time

A visual inspection of FLW values showed that the data was slightly skewed to the right. To better follow a normal distribution, we log-transformed FLW. The residual values obtained from the regression model using log-transformed FLW were normally distributed based on a visual quantile-quantile plot inspection and a Shapiro-Wilk normality test (*P* = 0.41) (Supplemental Fig. 1A and 1B).

**Fig. 2:**
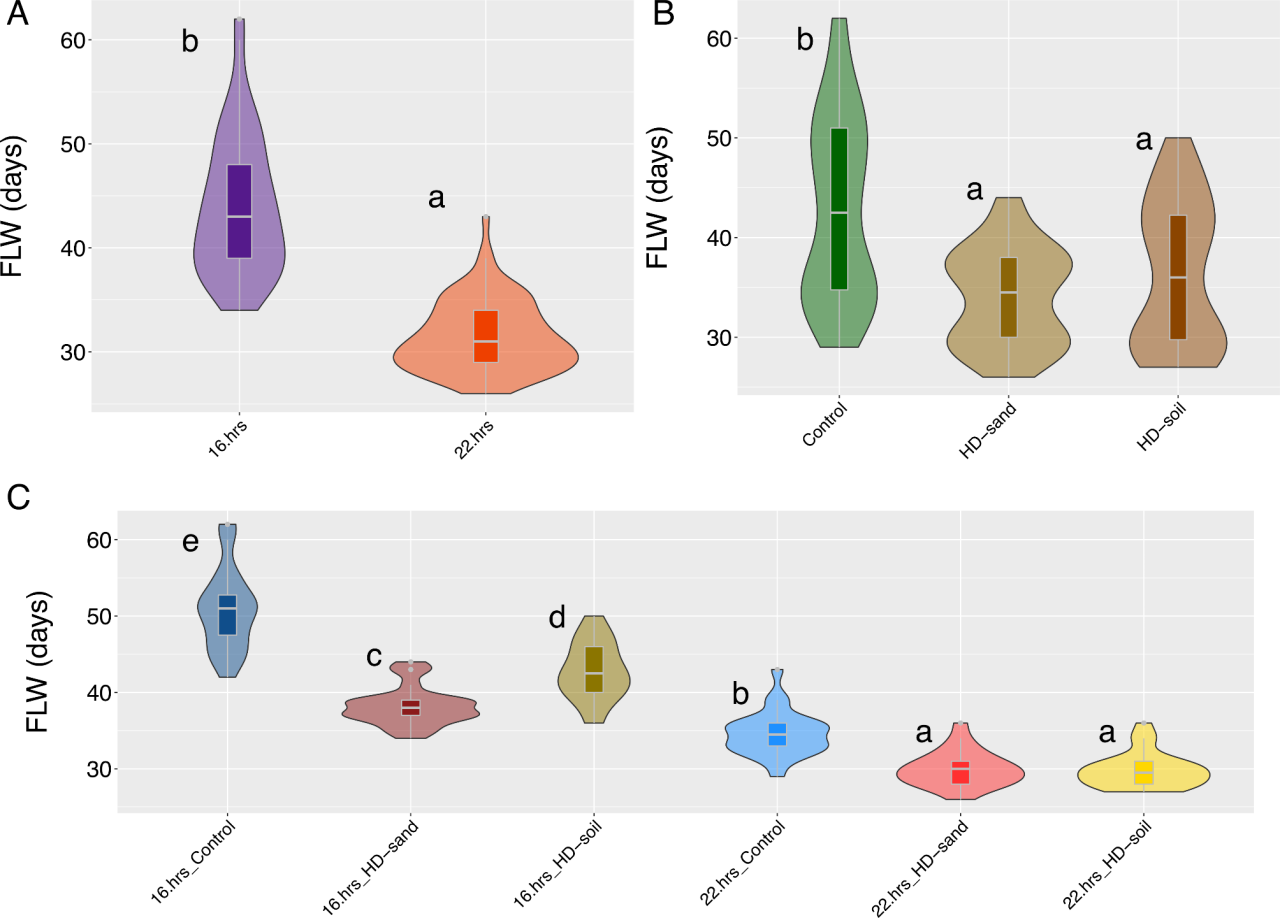
Effect of lighting and media/seeding-density in flowering time. **(A)** Effect of 16 and 22-hrs.day length on flowering time. **(B)** Effect of media/seeding-density treatments, control, HD-sand, and HD-soil on flowering time. **(C)** Effects of day length and media/seeding-density combinations on flowering time. Different letters represent significant differences (*P < 0.05*) of a Tukey HD test between treatments

Back-transformed FLW values across all treatments ranged from 26 to 62 days (Supplemental Table 2). Broad sense heritability for FLW was 0.87. Our analysis of variance (ANOVA) for log-transformed FLW identified significant differences (*P* < 0.01) among genotypes, media/seeding-density treatments, light treatments, blocks within light treatments, and the interaction between light and media/seeding-density treatments (Supplemental Table 3). We did not observe significant differences (*P* < 0.01) between the interaction of genotypes, light, and media/seeding-density treatments (Supplemental Table 3). By increasing lighting from 16 to 22-hrs., we detected, on average, a decrease of 12 days in FLW from 44 to 32 days (Fig. 2A). Planting at HD-soil decreased FLW time by six days compared to the Control (43 to 37 days), and HD-sand decreased FLW time by eight days compared to the Control (43 to 35 days) (Fig. 2B). When we grouped light and media/seeding-density treatments, we noticed, on average, that the plants that were grown at 22.hrs_HD-soil had the shortest FLW (29.7 ± 2.3), followed by 22.hrs_HD-sand (29.9 ± 2.9), 22.hrs_Control (34.6 ± 2.9), 16.hrs_HD-sand (38.2 ± 2.5), 16.hrs_HD-soil (42.7 ± 3.7), and finally 16.hrs_Control (50.8 ± 5.3) (Fig. 2C).

We performed a Tukey’s HSD multiple comparisons test among groups defined by the interaction between light and media/seeding-density. We detected five distinct and significantly different (*P* < 0.01) groups labeled as ‘a,’ ’b,’ ‘c,’ ‘d,’ and ‘e’ (Fig. 2C). Group ‘a’ showed the shortest average mean for FLW and included the treatments 22.hrs_HD-soil and 22.hrs_HD-sand. Following from shortest to longest FLW was group ‘b’ with the 22.hrs_Control treatment, then group ‘c’ with 16.hrs_HD-sand, group ‘d’ with 16.hrs_HD-soil, and lastly, group ‘e’ with 16.hrs_Control (Fig. 2C).

### The effect of lighting and media/seeding-density on plant height

**Fig. 3:**
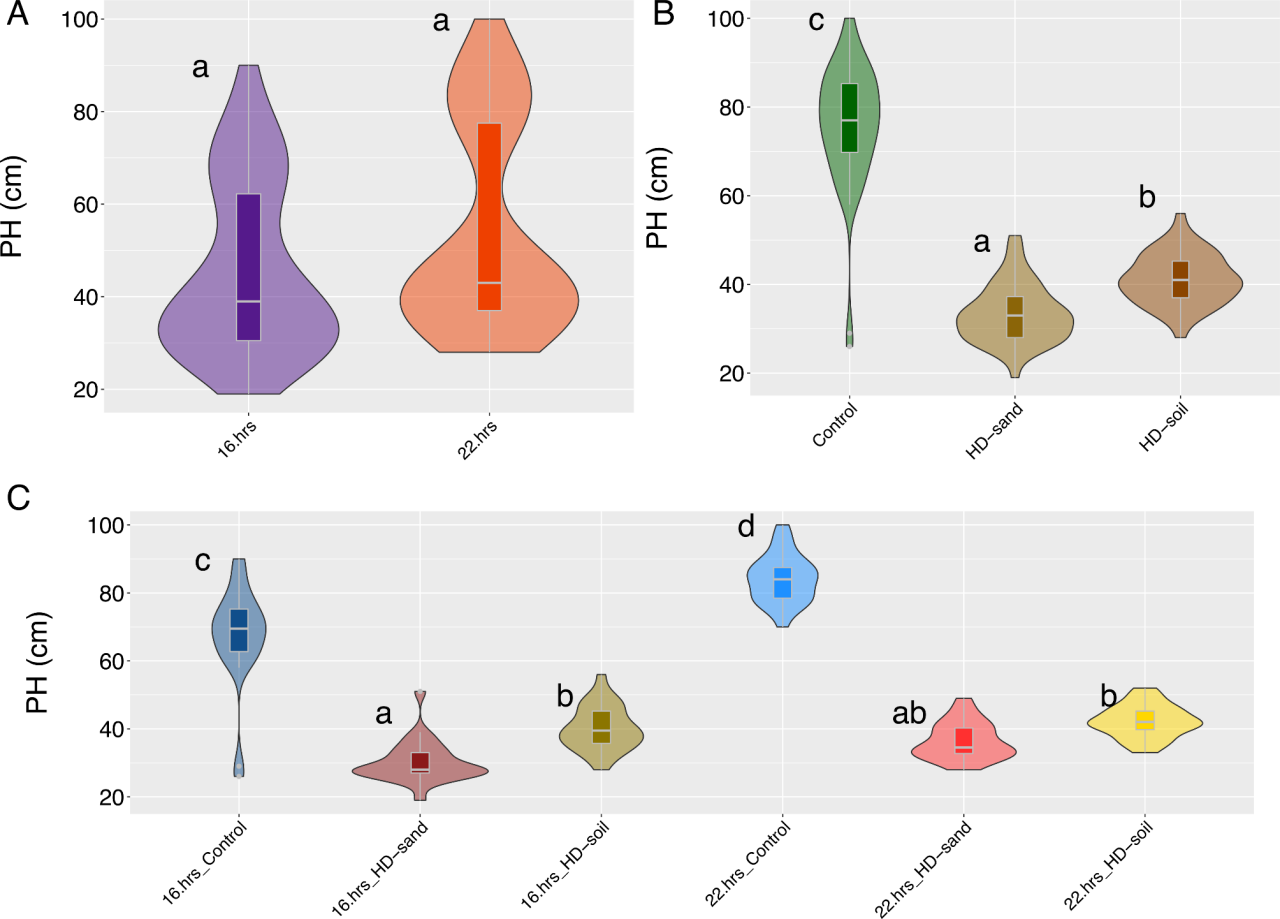
Effect of lighting and media/seeding-density in plant height. **(A)** Effect of 16 and 22-hrs. day length on plant height. **(B)** Effect of media/seeding-density treatments, control, HD-sand, and HD-soil on plant height. **(C)** Effects of day length and media/seeding-density combinations on plant height. Different letters represent significant differences (*P < 0.05*) of a Tukey HD test between treatments

A visual inspection of PH values showed that the data followed a normal distribution. The residual values obtained from the regression model using PH were normally distributed based on a visual quantile-quantile plot inspection and a Shapiro-Wilk normality test (*P* = 0.23) (Supplemental Fig. 1C and 1D). Plant height (PH) across all treatments ranged from 19 to 100 cm (Supplemental Table 2). In this study, the broad sense heritability we estimated for PH was 0.43. We conducted an ANOVA for PH and identified significant differences (*P* < 0.01) among media/seeding-density treatments, light treatments, and the interaction between light and media/seeding-density treatments (Supplemental Table 3). Planting at HD-soil decreased PH by 35 cm compared to Control conditions (from 76 to 41 cm), and HD-sand decreased PH by 43 cm (from 76 to 33 cm) (Fig. 3B). We grouped light and media/seeding-density treatments and observed that, on average, plants that were grown at 16.hrs_HD-sand had the shortest PH (30.2 ± 6.2), followed by 22.hrs_HD-sand (36.4 ± 5.3), 16.hrs_HD-soil (40.1 ± 6.5), 22.hrs_HD-soil (42.2 ± 4.7), 16.hrs_Control (67.4 ± 14.8), and finally 22.hrs_Control (84.1 ± 7.1) (Fig. 3C). A Tukey’s HSD multiple comparisons test among groups defined by the interaction between light and media/seeding-density showed four groups significantly different (*P* < 0.01) (Fig. 3C). The plants we grew at 16. hrs-HD-sand and 22.hrs_HD-sand were part of group ‘a’, followed by the 16. hrs-HD-soil and 22.hrs_HD-soil group in group ‘b’, 16.hrs-Control in group ‘c’ and finally group ‘d’ with 22.hrs-Control (Fig. 3C).

### The effect of lighting and media/seeding-density on the number of plants with a single seed

**Fig. 4:**
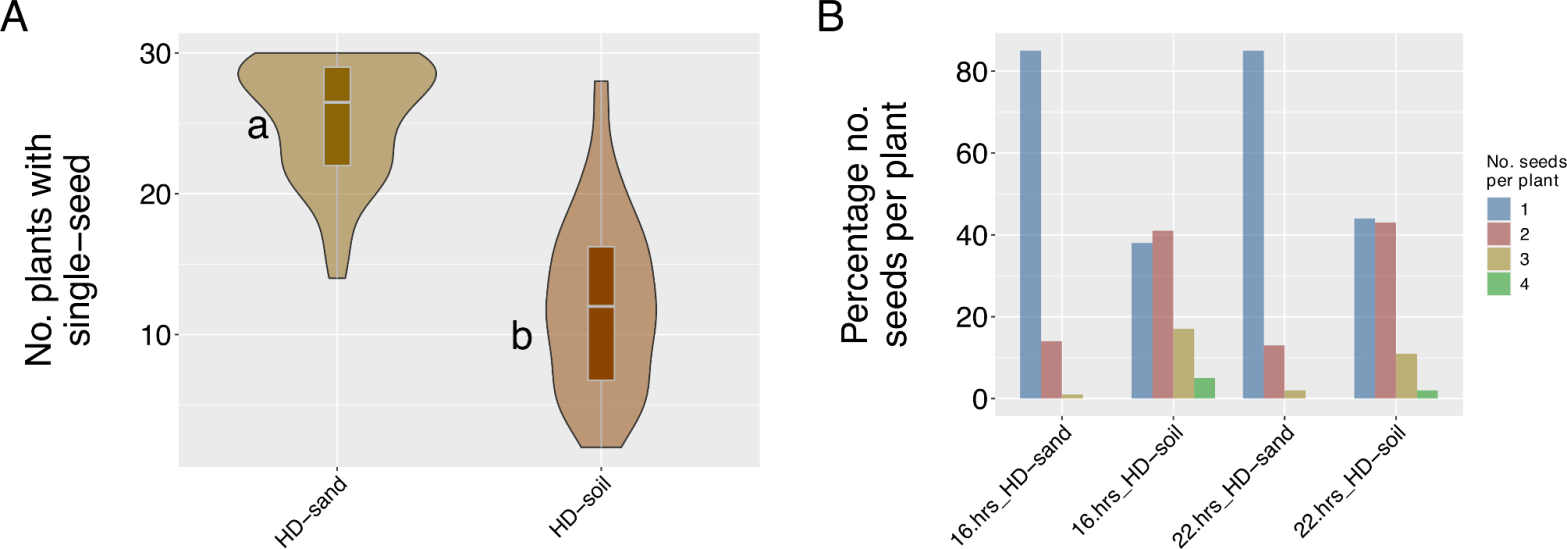
Effect of lighting and media/seeding-density on the number of plants with a single seed. **(A)** Average number of plants with a single seed from a sample of 30 plants collected on each HD-sand and HD-soil pot. **(B)** Percentage of plants with a single seed from a random sample of 30 plants collected in each pot grouped by lighting and media/seeding-density treatments. Different letters represent significant differences (*P < 0.05*) of a Tukey HD test between treatments

Across high-density seeded treatments, we randomly sampled 30 plants and observed a range between 2 and 30 plants having a single seed (Supplemental Table 2). We estimated that the number of plants with a single seed had a broad sense heritability of 0.47. The ANOVA we conducted identified that the type of media/seeding-density treatments showed significant differences (*P* < 0.01) for the number of plants with a single seed (Supplemental Table 3). Around 85% of the plants (with a mean of 25.4 over 30) we grew in HD-sand produced a single seed. In contrast, around 41% of the plants (with a mean of 14.4 over 30) we grew in HD-soil produced a single seed (Fig. 4A). When we parsed the data by light and media/high-seeding treatments and calculated the percentage of plants with 1, 2, 3, or four seeds, we observed that treatments planted in HD-sand either under 16 or 22-hrs. had, on average, 85% of the plants with one seed, 14% of the plants had two seeds and less than 1% had three seeds (Fig. 4B, Supplemental Table 4). We observed that plants planted in HD-soil, either under 16 or 22-hrs., had, on average, 41% of the plants with a single seed, 42% with two seeds, and 17% with more than two seeds per plant (Fig. 4B, Supplemental Table 4). We counted the total number of seeds harvested in pots with sand media seeded at high density, and on average, 126 seeds were harvested, representing 84% of the original 150 seeds seeded, showing 26% of barrenness or no germination in sand media under high-density treatments (Supplemental Table 1). On average, we observed that under Control conditions (low-density planting in pot mix), a panicle produced 20 seeds.

## Discussion

Our results showed that supplemented light and seeding density can significantly decrease flowering time in an additive fashion when applied to spring oats that are grown in greenhouse conditions. González-Barrios et al. [19] showed that increasing supplemented light in oats from 16 to 22-hrs. decreased flowering time by 11 days. Our results showed that increasing supplemental light from 16 to 22-hrs. had the most considerable effect and decreased flowering time by 12 days on average. These results are comparable to those reported by González-Barrios et al. [19]. In addition, we observed that the seeding density can significantly decrease flowering time. Planting at high density reduced the flowering time by seven days compared to control conditions. Collard et al. [8] reported that rice grown under limited media and space constraints significantly flowered earlier than rice grown under normal conditions. When sand was used as growing medium, we observed significant differences compared to potting-mix when plants were grown using 16-hrs. of supplemented light. These results correspond with the findings of Cisar et al. [18], in which coupling high plant density with limited soil fertility and moisture levels in sand conditions effectively decreased flowering time in oats. Our results showed that the differences in FLW between plants grown at high density in potting-mix and sand disappeared when plants were provided 22-hrs. of light. When we combined speed breeding methodologies [9, 17, 20] with high-density planting [18], we could decrease flowering time in an additive fashion by 20 days on average, from 50 days under control conditions to 30 days under space constraint and 22 h. a day length.

We evaluated plant height as a proxy to determine the likelihood of experiencing lodging. We observed significant differences between media-type-seeding treatments. Plants grown at low densities were significantly taller than those at high-densities. We observed a small but significant difference between plants grown in the sand and potting-mix at high-densities. The reduced amount of nutrients and lower water retention could explain the shorter stature of plants grown in sand media compared to the potting-mix, as reported by Cisar et al. [18]. Despite these differences, no lodging was observed in either media. Our results demonstrated that plants did not need external support when grown in our speed-breeding conditions, simplifying care and handling labor during rapid-generation-advancement stages.

As reported by Cisar et al. [18], we observed that growing spring oat plants at high-density in sand media significantly produced a higher proportion of plants that developed a single seed. About 85% of the plants in sand media yielded a single seed, the remaining 14% produced two. In contrast, when we grew plants at high-density in potting-mix media, only 41% of the plants developed a single seed. Interestingly the light treatment had no effects on the number of plants that produced a single seed. The combination of high-density planting, low nutrients, and water-stressed induced by the sand media was suggested by Cisar et al. [18] as the cause of high-frequency plants with a single seed. To follow SSD assumptions, sand must be used as the growth media. This treatment does generate barrenness in about 26% of the plants. This barrenness value corresponds with Cesar et al. [18] observations. Their study analyzed the effect of barrenness in the mean and genetic variance of yield, oil content, plant height, and flowering time in families grown in the sand at high seeding densities and under standard SSD techniques. Their results showed no selective elimination of genotypes for any trait with non-significant comparisons between density means and density variances [18]. This percentage of barrenness must be considered during the population development to account for the number of plants with no seeds and obtain the final population desired at the end of the rapid-generation-advancement process.

Breeding of new, advanced cultivars can take several years. Following the crossing of selected parents, 4–6 generations of inbreeding are typically required to develop genetically stable lines [9]. This process can be particularly time-consuming for field crops in which only 1 or 2 generations per year can be achieved [20]. Our new Single-Seed-SpeedBulks protocol for spring oats combines the time-saving advantages of speed breeding [9, 19, 20] with the decrease in space and labor of the modified SSD method proposed by Cisar et al. [18]. This protocol can cost-effectively generate breeding populations, recombinant inbred lines, and nested association mapping lines in spring oats. We created a Standard Operation Procedure (SOP) (Supplemental File 1) describing in detail how to execute this protocol and combine it with a de-bulking strategy to advance lines from crossing to seed-increased F_4_ lines plots in one year. This protocol or parts may be used in other spring small grains to improve rapid-generation advancement and decrease cost and labor.

### Electronic supplementary material

Below is the link to the electronic supplementary material.

Supplemental Fig. 1. Log-transformed flowering time residual distributions (A), and quantile-quantile plot. Plant height residuals distributions (C), and quantile-quantile plot (D)

Supplemental Table 1. Description of oat varieties and raw phenotypic data collected in this study.

Supplemental Table 2. Summary of flowering time, plant height, and the number of plants with a single seed phenotypic data

Supplemental Table 3. Analysis of variance table of log-transformed flowering time, plant height, and the number of plants with a single seed

Supplemental Table 4. Percentage of plants with 1, 2, 3, or > 4 seeds when grown at 16 or 22 h. of light treatment in soil, or sand media

Standard Operating Procedure: ‘Single-Seed-SpeedBulks:’ a protocol that combines ‘speed breeding’ with a cost-efficient modified single-seed descent method for rapid-generation-advancement in oat (*Avena sativa* L.)

## Data Availability

All datasets supporting the conclusions of this article are included within the article and in its Supplementary Tables and File.

## Abbreviations

SOP: Standard Operation Procedure
SSD: Single-Seed-Descent
RGA: Rapid Generation Advance
mSSD: modified SSD
UIUC: University of Illinois at Urbana-Champaign
PCF: Plant Care Facility
FLW: Flowering Time
PH: Plant Height
ANOVA: Analysis of Variance
