# Supplementary figures and images for: ‘Single-Seed-SpeedBulks:’ a protocol that combines ‘speed breeding’ with a cost-efficient modified single-seed descent method for rapid-generation-advancement in oat (Avena sativa L.)
Source: Plant Methods. 2023 Aug 27;19:92. doi: 10.1186/s13007-023-01067-1 (PMC10464167; doi:10.1186/s13007-023-01067-1)

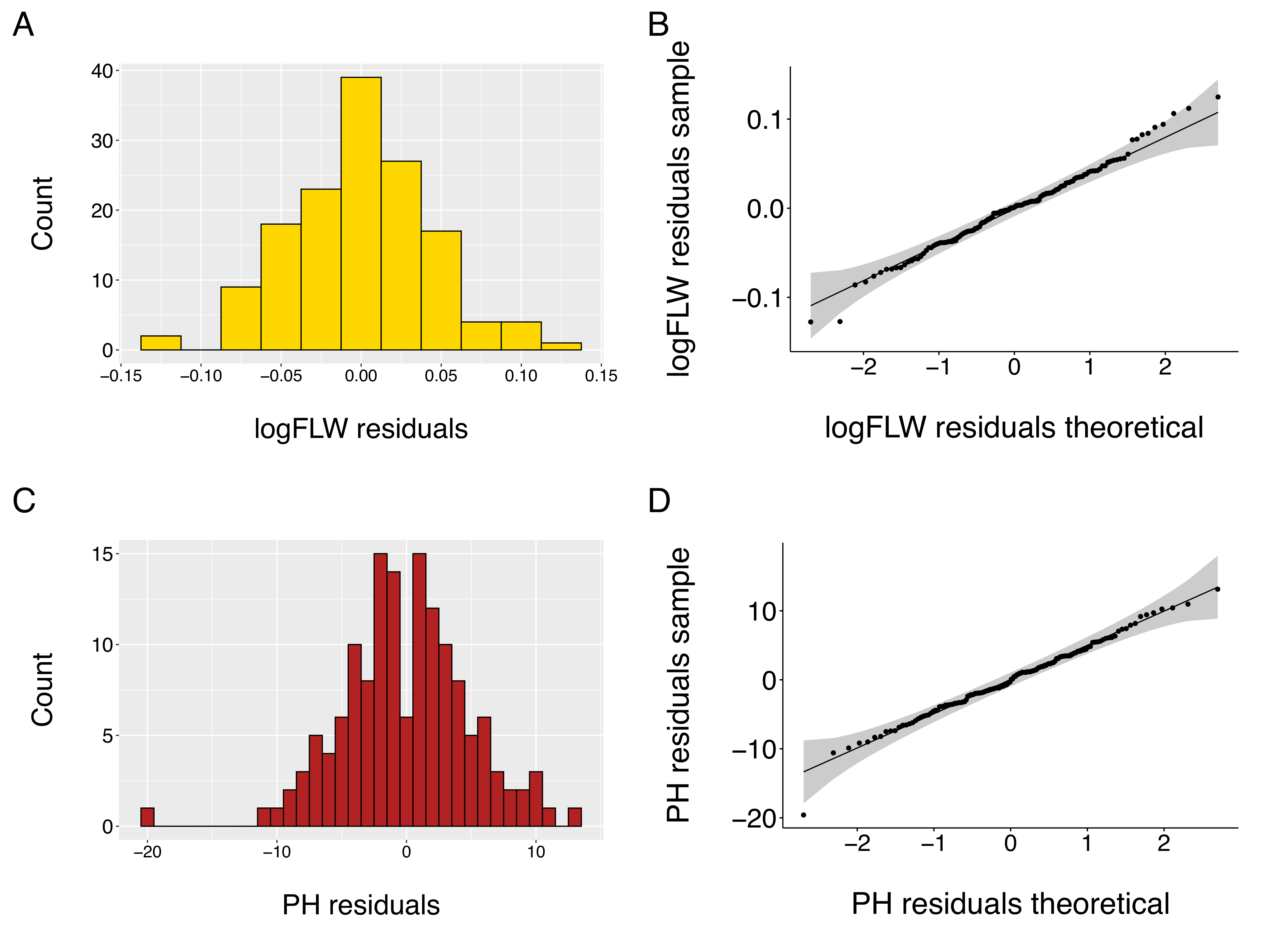

Supplement: Supplementary file 1 — Supplemental Fig. 1. Log-transformed flowering time residual distributions (A), and quantile-quantile plot. Plant height residuals distributions (C), and quantile-quantile plot (D) [file 13007_2023_1067_MOESM1_ESM.tif]
