# Supplementary material for: ‘Single-Seed-SpeedBulks:’ a protocol that combines ‘speed breeding’ with a cost-efficient modified single-seed descent method for rapid-generation-advancement in oat (Avena sativa L.)
Source: Plant Methods. 2023 Aug 27;19:92. doi: 10.1186/s13007-023-01067-1 (PMC10464167; doi:10.1186/s13007-023-01067-1)
Supplement: Supplementary file 6 — Standard Operating Procedure: ‘Single-Seed-SpeedBulks:’ a protocol that combines ‘speed breeding’ with a cost-efficient modified single-seed descent method for rapid-generation-advancement in oat (Avena sativa L.) [file 13007_2023_1067_MOESM6_ESM.pdf]

Standard Operating Procedure: 'Single-Seed-SpeedBulks:' a protocol that combines 'speed breeding' with a cost-efficient modified single-seed descent method for rapid-generation-advancement in oat (*Avena sativa* L.)

Milcah Kigoni. First author**.** Department of Crop Sciences, University of Illinois, Urbana-Champaign, Turner Hall AW-101, 1102 S. Goodwin Ave., 046, Urbana, IL 61801, USA. Email: mkigoni2@illinois.edu

Megan Choi. Department of Crop Sciences, University of Illinois, Urbana-Champaign, Turner Hall AW-101, 1102 S. Goodwin Ave., 046, Urbana, IL 61801, USA. Email: meganchoi8@gmail.com

Juan David Arbelaez. Corresponding author**.** Department of Crop Sciences, University of Illinois, Urbana-Champaign, Turner Hall AW-101, 1102 S. Goodwin Ave., 046, Urbana, IL 61801, USA. Email: arbelaez@illinois.edu

1.0 Introduction

The purpose of this protocol is to describe the requirements and procedure of rapidly advancing the breeding cycle of Spring Oat (*Avena sativa*) between F_2 and F_6 in one year by combining the Single-Seed-SpeedBulks protocol with a Tray-Single-Seed-Descent (Tray-SSD) method.

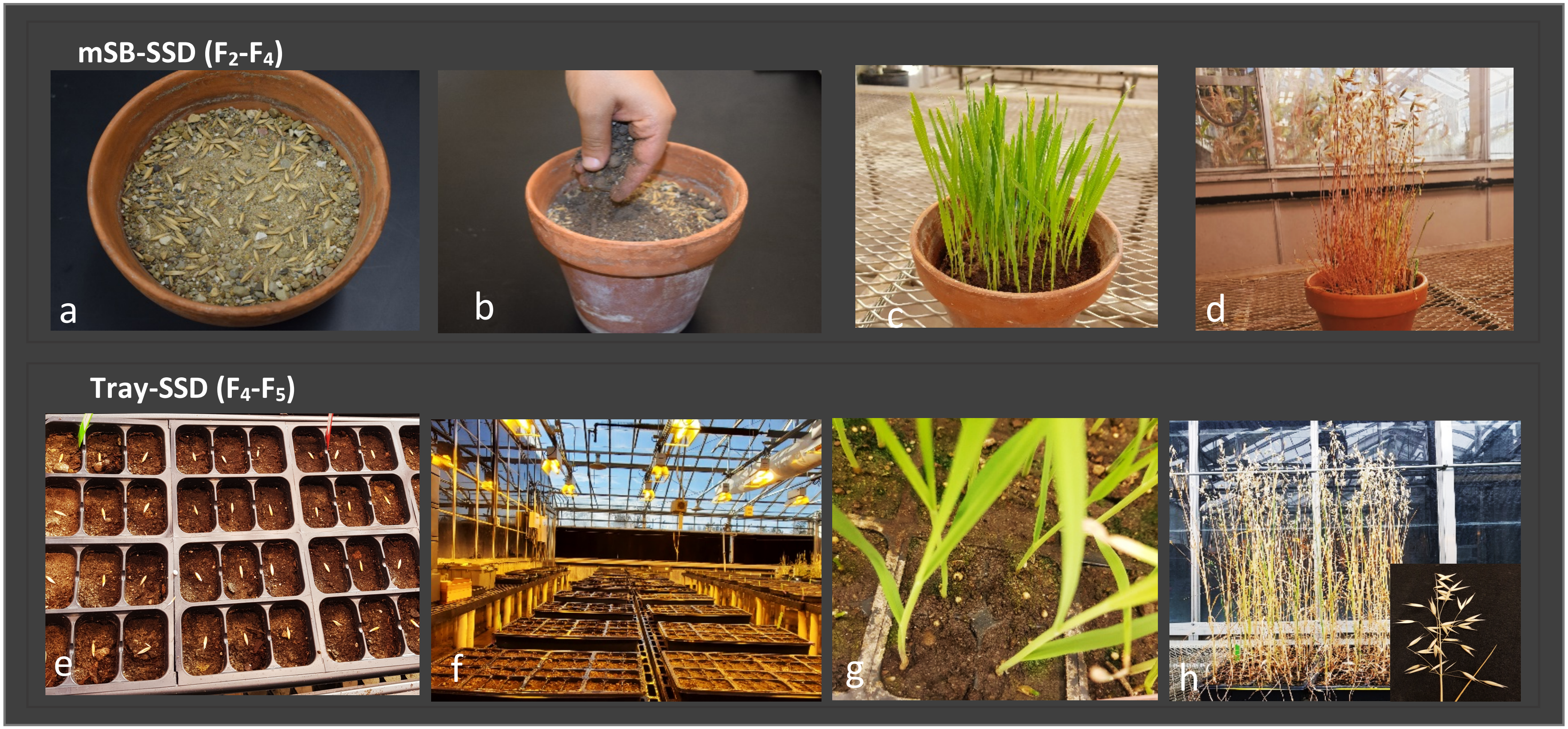

Figure 2: A step-by-step overview of the 1-year Spring oat rapid generation advancement protocol. a) fill pot with washed torpedo sand and place 120 seeds on the sand media, b) cover seeds with potting mix soil media c) grow the plants under 22hr light and water regularly d) harvest the seeds per pot in bulk at maturity. Repeat this mSB-SSD process from F_2-F_4. e) fill seeding trays (4 X 12) with potting soil media and plant each seed from the harvested F_4 bulk seeds in an individual cell, f) fertilize and water as needed and place the trays under 22hr light. g) after 3 weeks thin the trays to leave 1 plant per cell, h) harvest each panicle of F_5 separately at maturity. These panicles are threshed and planted in the field as panicle rows (stage 1 evaluation).

1.1 Greenhouse Facility Requirements

This protocol is fully implemented in a greenhouse conditions fitted with 22hr supplemental lights, and programmable Day/Night light intensity and Heat/Vent temperature controls. Alternative options provided by Ghosh et al. 2018 are recommended for site-specific growth conditions optimization.

2.0 mSB-SSD Procedure

This procedure is conducted for two generations: F_2 to F_4 and F_4 to F_5

**Materials**	**Germplasm**<br>● F_2 and F_4 seed<br>**Growth medium**<br>● Torpedo sand<br>● Weedmix soil comprised of one part peat, one part soil, and one part torpedo sand<br>**Planting container**<br>● six-inches-clay-pots or similar size alternatives<br>**Seeding density**<br>● 120-150 seeds per pot
**Planting**	● Fill in the pots with 2kg of Torpedo sand per pot<br>● Evenly place 120 seeds on the sand media<br>● Cover the seeds with 0.5kg of the Weedmix soil<br>● Water the pots with a gentle sprinkle<br>● Arrange pots on a bench ensuring each pot is under direct under 22hr light
**Plant care**	**Watering**<br>● Water the pots daily or as needed between Feekes 1-11<br>● End watering about 5 days to harvest<br>**Fertilizing**<br>● Do not apply fertilizer to the plants<br>**Plant health**<br>● Heat stress may occur due to increased temperature from prolonged greenhouse lamps – room temperature conditions can be adjusted if extreme heat stress is observed.

	• Thrips and Aphids are a great risk especially at Feekes 8-10.5, they affect plant health and successful seed set<br>• Lodging may be observed in some cases which may be due to the frequency of fertilization, this may be adjusted as needed
**Harvesting**	• At maturity, harvest the pots by cutting the bulk of plants per pot using scissors.<br>• place the bundles per pot in respective labelled bag.

3.0 Tray-SSD Procedure

This procedure is conducted for F_4 to F_5 generation

**Materials**	**Germplasm**<br>• F_4 seed<br>• At least 2 check varieties (important for troubleshooting method vs germplasm issues)<br>**Growth medium/soil**<br>• Weedmix soil comprised of one part peat, one part soil, and one part torpedo sand<br>**Planting container**<br>• 48-cell trays of 5.8X4cm cell size and flats with holes<br>**Seeding density**<br>• 1 seed per cell<br>**Fertilizer**<br>• Liquid fertilizer – 150 p.p.m blend of Nitrogen (20%), Phosphorous (20%) Potassium (20%)
**Planting**	• Place trays on a potting bench and fill in the cells with Weedmix soil leaving 1.5 inches space at top of each cell<br>• Water the trays to dampen the soil and place a labelled stake to mark beginning of each family in a tray<br>• Plant a single seed at the center of each cell<br>• When all cells are filled cover the cells with soil leaving about 0.5-inch space at top to ensure the cells remain distinguishable<br>• Water the trays with a gentle sprinkle

	● Adjust tray position on bench to ensure they are under direct light
**Plant care**	**Watering**<br>● Feekes 1: top watering (sprinkle)<br>● Feekes 2-11: gently water the trays from the side or the bottom if hole-less flats are used<br>● End watering about 3 days to harvest<br><br>**Fertilizing**<br>● Apply the liquid fertilizer to the soil once a week between Feekes 9-10.5<br>**Thinning**<br>● Use scissors to thin the trays at the end of Feekes 1 or after 3 weeks to leave a single plant per cell
**Harvesting**	● At maturity, harvest the trays by cutting each panicle with scissors and placing them in respective labelled bag.

4.0 Risks and Troubleshooting

- Heat stress may occur due to increased temperature from prolonged greenhouse lamps – room temperature conditions can be adjusted if extreme heat stress is observed.
- Thrips and Aphids are a great risk especially at Feekes 8-10.5, they affect plant health and successful seed set
- Lodging may be observed in some cases which may be due to the frequency of fertilization, this may be adjusted as needed.
- Due to variation in greenhouse conditions, lighting sources, soil and other growth conditions across breeding programs and locations, we recommend running a test growth cycle with the above protocol, to optimize it as needed prior to implementing it with the breeding material.

5.0 References

Ghosh, Sreya et al. 2018. "Speed Breeding in Growth Chambers and Glasshouses for Crop Breeding and Model Plant Research." *bioRxiv* 13(December): 369512.
